# Persistent elevation of aspartate aminotransferase in a child after incomplete Kawasaki disease: a case report and literature review

**DOI:** 10.1186/s12887-020-1975-8

**Published:** 2020-02-17

**Authors:** Pejman Rohani, Farid Imanzadeh, Aliakbar Sayyari, Maryam Kazemi Aghdam, Reza Shiari

**Affiliations:** 1 Pediatric Gastroenterology, Hepatology and Nutrition Research Center, Research Institute for Children Health, Shahid Beheshti University of Medical Sciences, Shariati Ave, in front of Hoseinieh Ershad, Tehran, Iran; 2 Pediatric Pathology Research Center, Research Institute for Children Health, Shahid Beheshti University of Medical Sciences, Tehran, Iran; 3 Pediatric Research Institute for Children Health, Shahid Beheshti University of Medical Sciences, Tehran, Iran

**Keywords:** Kawasaki disease, Macro-AST, Aspartate aminotransferase, Children, Pediatric, Liver, Case report

## Abstract

**Background:**

Interpretation of abnormalities in liver function tests, especially in asymptomatic children, is a common problem faced by clinicians. Isolated elevation of aspartate aminotransferase may further puzzle physicians. Macro-aspartate aminotransferase (AST) results from complexes AST produces with other plasma components, such as immunoglobulin. To our knowledge, this is the first report on a case of macro-AST-associated incomplete Kawasaki disease (KD). It is to make physicians aware of this benign condition and help to prevent extensive, unnecessary investigations and invasive workups.

**Case presentation:**

A 16-month old boy with a 7-day history of fever was admitted to our pediatric ward for pyrexia workup. After complete investigations, KD was confirmed by a pediatric rheumatologist. During his admission and serial follow-up tests, an isolated AST elevation was noted. Comprehensive tests were performed and using the polyethylene glycol (PEG) precipitation method, macro-AST was confirmed. The patient has been followed up for 3 years, and so far, the benign nature of this condition has been confirmed.

**Conclusion:**

Clinicians should consider testing for macro-AST when elevated AST is the only abnormal lab finding. Although an uncommon finding, macro-AST may be seen in both children and adults. There are many reasons for this phenomenon, including resolved acute hepatitis or in some cases, inflammatory bowel disease, hepatic malignancy, monoclonal gammapathy, celiac disease, or KD; however, it may be observed in asymptomatic healthy children as well. Using the PEG precipitation method, a definitive diagnosis can be made. In none of these conditions does macro-AST have any prognostic significance. An appreciation of macro-AST may prevent the need for more invasive investigations to which patients may be unnecessarily subjected. It is important to recognize this condition as benign and assure patients that no specific treatment is required.

## Background

Interpretation of liver function abnormalities is a common problem faced by clinicians. Abnormal liver function tests in asymptomatic children, not only pose a challenge to physicians, but may also be worrisome for the child’s family. Elevated liver enzymes may be benign, but physicians often perform extensive investigations which include workup for liver, hematologic and bone diseases.

Macro-aspartate aminotransferase (macro-AST) is a condition where AST binds to macromolecules such as immunoglobulins or other plasma components. Due to their large size and mass, macro-AST complexes are not easily cleared by the renal glomeruli, resulting in elevated serum levels. The described macromolecules comprise of macro-amylase, macro-prolactin, macro-LDH and macro-alkaline phosphatase [1]. This condition is observed in hepatitis C virus [2], monoclonal gammopathy and malignancy patients [3] and normal children [4]. We reviewed the literature and reported a child with Kawasaki disease (KD) and macro-AST.

## Case presentation

A 16-month old boy with a 7-day history of fever was admitted to the pediatric ward for fever work up. Three days prior to his admission, he had suffered from bilateral bulbar, nonpurulent conjunctivitis. Prior to his admission, the patient had also been treated with two courses of oral antibiotics, azithromycin and amoxicillin with clavulanic acid. On examination, there were no signs of coryza or upper respiratory tract infection. The oral and pharyngeal mucosa were erythematous and there was unilateral cervical lymphadenopathy (measuring 1 × 1.5 cm). Abdominal exam was unremarkable. Abdominal ultrasound revealed normal liver, biliary system and spleen, and echocardiogram was normal. Blood and urine test results are summarized in Table 1.

**Table 1:** Lab results of first admission and follow up visits

	Pre IVIG Treatment	Two weeks from the onset	Three months from the onset	Six months from the onset	Nine months from the onset	Nine months from the onset	Nine months from the onset
WBC 10^3^/micl	15.1	9.6	—	—	—	—	—
RBC 10^6^/micl	3.8	4.11	—	—	—	—	—
Hemoglobin g/dl	9.8	11	—	—	—	—	—
MCV fl	78.8	79	—	—	—	—	—
Platelet 10^3^/micL	519	362	—	—	—	—	—
ESR mm/hr	60	5	—	—	—	—	—
CRP mg/L	45	1	—	—	—	—	—
CPK mcg/L	150	183	94	98	92	99	70
LDH IU/L	417	309	268	284	280	283	191
AST U/L	351	252	198	205	230	212	197
ALT U/L	40	17	11	12	15	15	17
ALP U/L	485	213	215	190	204	201	313
GGT U/L	30	13	8	7	10	12	9
ALB g/dl	4	4.4	4	4.2	4.8	4.7	3.9
Total Protein g/dl	7	6.9	6	6.4	6.9	6.8	6.3
PT Sec/INR	13/1	13.1	13/1	13/1	13.5/1.1	13/1	12/1
PTT Sec	37	37.5	38	38.5	39.6	37	35
Other complementary test							
ANA	Neg	HBS Ag	Neg	Retic	2.2%		
Anti LKM	< 1.0 Ru/ml	IgM Anti HBC	Neg	Coombs	Neg		
Anti Smooth Muscle Ab	Neg	Anti HCV	Neg	Indirect Coombs	Neg		
Anti TTG IgA	< 1 Ru/ml	Anti HIV	Neg	G6PD	Sufficient		
IgA	137 mg/dl	Ammonia	0.89 Microg/ml	Hemoglobin A	95.2%		
Ceruloplasmin	39 mg/dl	Lactate	21.3 mg/dl	Hemoglobin A2	3%		
Alfa 1 Antitrypsin	151 mg/dl	Triglycerides	58 mg/dl	Hemoglobin F	1.8%		
TSH	10.22 micg/dl	Cholesterol	163 mg/dl	Ferritin	27.78 ng/ml		

Constellations of prolonged fever (more than 7 days), bilateral bulbar, nonpurulent conjunctivitis, unilateral cervical lymphadenopathy, erythematous oral and pharyngeal mucosa, erythrocyte sedimentation rate = 60, c-reactive protein = 45, anemia (Hg = 9.8), thrombocytosis (PLT = 519,000), leukocytosis (WBC = 15,100), negative urine and blood culture and history of complete vaccination (measles, mumps, and rubella [MMR] at the end of age 1) fulfilled the diagnosis of incomplete KD according to the American Heart Association (AHA) guidelines.

Once the diagnosis of atypical KD was made, the patient was commenced on intravenous immunoglobulin (IVIG) therapy. Treatment protocol was 2 g/kg of IVIG over 12 h and 35 mg/kg of aspirin divided q6h for 48 h. According to the standard protocol, high-dose aspirin (80–100 mg/kg) should reduce to 30–40 mg/kg in patients with elevated liver enzymes. After 36 h, the patient’s fever subsided. Aspirin dose reduced to 3 mg/kg once daily orally until 6 wk. after illness onset. An echocardiogram was repeated in 2 weeks, which revealed normal results. In a follow-up visit, lab data showed normal blood parameters, except for a persistent AST elevation. The patient was subsequently referred to a hepatologist and a hematologist for further investigations (Table 1).

The patient was seen by the hepatologist after 3 months. The patient was in good general health. The full physical examination was within normal limits and there were no signs or symptoms of liver disease. Further follow-up lab data and serial follow up tests were obtained in three to nine-month intervals. The results are summarized in Table 1.

Following 3 years of observation, the final investigation was performed by the polyethylene glycol (PEG) precipitation method. Using this test, we can differentiate macro-AST from other conditions with elevated AST. This is the best screening test for macro-AST detection [5]. The best concentration of PEG solution is 25% (m/v) (5). In this method, we added 0.2 ml of patient’s serum to an equal volume of PEG 6000 (Merck ART 807491). After 5 min at room temperature, the serum was centrifuged for 30 min at 3000 rpm, and the clear supernatant was sent for measuring AST. The same process was performed for two control samples. Baseline AST level and post-PEG effects are shown in Table 2. PEG perceptible activity (%PPA) is calculated as 100x [(AST activity – AST activity +PEG)/AST activity] [5, 6]. The results are shown in Table 2. According to the literature, a threshold of 73% for PPA is standard for the diagnosis of macro-AST, and in our patient %PPA was 80% [5, 7, 8].

**Table 2:** PEG perceptible activity (%PPA)

	AST level	AST level + PEG	%PPA
Index case	197	38	80
Control 1	108	89	17
Control 2	462	300	35

## Discussion

AST is an enzyme found predominantly in the liver and heart; however, it is also present in skeletal muscles, brain, pancreas, RBC, WBC, lungs and kidneys. There are two similar isoenzymes of AST localized in the cytoplasm and mitochondria and are encoded by *GOT1* and *GOT2* on chromosomes 10q24 and 16q12, respectively [9]. AST catalyses the transfer of an amino group to α-Ketoglutaric acid to make glutaric acid [9]. AST rises 12 h after hepatic damage, with peak levels seen at 24 to 48 h [9]. Isolated elevated AST, especially in asymptomatic children, poses a challenge to clinicians, resulting in often extensive, costly investigations. It is difficult to know the best investigative approach to these children. Some of the most important differential diagnoses and investigations that would aid in the differentiation of possible causes for increased AST are listed in Table 3.

**Table 3:** Different causes of elevated level of AST and diagnostic work-up

Cause	Tests
Hepatocellular disease	ALT, ALP, GGT
Cardiac disease	CK MB
Hemolytic disease	LDH, Haptoglobin
Muscular disease	CPK, Aldolase
Medication	Erythromycin

Macro enzyme production phenomenon was suggested in 1964 [10]. There are a variety of macro enzymes such as amylase, lipase, alkaline phosphatase, gamma-glutamyltransferase, AST, lactate dehydrogenase and creatine kinase [11, 12]. Macro enzymes are divided to two groups [12]. High molecular weight complex is formed by self-polymerization or bonding with other molecules such as immunoglobulin [12–14]. Circulating autoantibody and serum enzyme build enzyme-immunoglobulin complexes [12]. These large molecules do not filter through the renal glomeruli [12, 15]. The definite mechanism of immunoglobulin-complex enzyme formation is not known, but the most probable mechanism is the antigen-driven theory [12].

Macro-AST was first recognized by Konttinen et al. in 1978 [16]. We used MEDLINE, PubMed, and Google Scholar databases to review the literature. The prevalence of macro-AST in either children or adults has not been established. Caropreso et al. performed a study to determine the reason for elevated isolated AST in a tertiary medical center and found that the prevalence of macro-AST was between 13.1 and 60% for adults and 38.6% for children [4]. There are many different diseases in adults linked to macro-AST in the literature (Table 4).

**Table 4:** Different causes of macro AST

**Normal child** [17]	
**Auto immune Disorder**	
Systemic lupus erythematosus [18]	
Rheumatoid arthritis [13, 18]	
Ankylosing spondylitis [18]	
Cryoglobulinemia [3, 18]	
Inflammatory bowel disease [2, 18, 19]	
Celiac disease [18, 20]	
Allergic injection immunotherapy [18, 21]	
**Liver Diseases**	
Hepatologic Malignancy [18, 22]	
Chronic Liver Disease [18, 22, 23]	
Chronic Hepatitis C [2, 24]	
Acute Hepatitis [25]	
**Drug** (Erythromycin) [26]	

When compared to adults, it may seem that isolated macro-AST is more often a benign phenomenon in children [4]. Kulecka et al. reported a heterozygous mutation in *GOT1* that is associated with familial macro-AST [27]. There are different ways for detecting macro molecules such as macro-AST. The gold standard is probably gel filtration chromatography, but it is not used routinely in clinical practice [28]. Ultracentrifugation is another method with high diagnostic validity [28]. The PEG precipitation method is a screening test, but it is not as sensitive as the previous methods described [5]. Proteins A and G, which are bacterial proteins, bind to human immunoglobulins (IgG) and have been used by van Wijk et al. as another diagnostic test for detecting macro-AST [28]. Baser et al. used another practical technique and kept plasma samples of eight patients refrigerated at 2–8 degrees Celsius for 5 days. They found that macro-AST molecules were not stable and decreased by 65% [29].

KD is a multisystemic disease. In spite of the self-limited nature of the acute illness, vasculitis has three distinct phases. The second chronic vasculitis phase may last weeks to years [30]. Gastrointestinal and hepatobiliary systems are involved in many patients and may sometimes be the first presentation of this disease [30]. The disease may present in various ways including: elevated liver enzymes, hydrops of gallbladder, acute cholecystitis, acute cholangitis, hepatitis and enterocolitis [31]. However, none of the aforementioned conditions cause chronic liver or gastrointestinal diseases and resolve following the acute disease process. According to the transformation-of-self antigen theory, as mentioned above, autoantibody formation against enzymes released from injured liver may happen [12]. The IgG is the most likely immunoglobulin that makes an immune complex with AST [25, 32]. Generally, in all diseases with macro enzyme elevation, the total immunoglobulin level is not increased. Elevated macro creatine kinase with IgA for 20 days was reported in a 13-month-old boy with KD by Inoue et al. [33]. In KD, as in other autoimmune diseases, transient elevation of IgG is expected and partial damage to some hepatocytes or biliary cells is probable [12, 25].

In addition, patients with KD are usually treated with intravenous immunoglobulin. Probably the increase in IgG level will allow for the production of immune complexes. According to the literature for persistent elevation of macro-AST, high degrees of inflammation are not mandatory for the diagnosis of this disease [12]. Macro-AST has been reported following self-limited acute hepatitis [25]. There are different ways to produce macro enzymes other than formation of immune complexes, particularly for enzymes such as GGT, ALK, and AST, which are located in hepatobiliary cell membranes [12]. These macro enzymes are found to be low in healthy individuals. According to one theory, detergent property of the bile separates macro enzymes and connects them to a lipoprotein molecule in the blood stream [34–36]. In another theory, plasma membrane fragments detached from the hepatobiliary system and moved in the blood stream like vesicles containing many enzymes [34, 37, 38]. All the above data show that macro-AST may be seen in KD. On other hand, we cannot conclude that KD was the cause of macro-AST in our patient. Macro-AST can be seen even in healthy asymptomatic children. It is not known how long patients with macro-AST should be followed up. According to a study by Caropreso et al., 1 to 16-year follow-up showed no significant change in the general health of patients [4]. A three-year follow up of our patient has shown that he is completely healthy, and confirmation of macro-AST revealed a benign course with good prognosis. A liver biopsy was not performed.

## Conclusion

Isolated elevation of AST is an uncommon finding in children, the etiology of which remains conspicuous. As with any illness, a complete history and physical examination should be undertaken. Blood sampling should follow the guideline suggested in Table 3. If all the aforementioned investigations are performed without a suitable explanation of the etiology, then macro-AST should be suspected. With a simple method such as PEG precipitation, a definitive diagnosis can be made. This approach may prevent performing extensive, costly and invasive workups.

## Data Availability

All data generated during this study are included in this publication [and its supplementary information files]. No data analysis was provided during this study.

## Abbreviations

ALT: Aspartate alanin transaminase
ANA: Anti nuclear antibody
Anti LKM: Anti liver kidney microsomal
Anti SMA: Anti smooth muscle antibody
Anti TTG: Anti tissue transglutaminase
AST: Aspartate aminotransferase
CPK: Creatinine phosphokinase
G6PD: Glucose 6 phosphate dehydrogenase
GGT: Gama glutamyl transferase
KD: Kawasaki disease
LDH: Lactate dehydrogenase
PEG: Polyethylene glycol
PPA: PEG perceptible activity
